# Amyloid precursor protein expression and processing are differentially regulated during cortical neuron differentiation

**DOI:** 10.1038/srep29200

**Published:** 2016-07-07

**Authors:** Petra Bergström, Lotta Agholme, Faisal Hayat Nazir, Tugce Munise Satir, Jamie Toombs, Henrietta Wellington, Joakim Strandberg, Thomas Olsson Bontell, Hlin Kvartsberg, Maria Holmström, Cecilia Boreström, Stina Simonsson, Tilo Kunath, Anders Lindahl, Kaj Blennow, Eric Hanse, Erik Portelius, Selina Wray, Henrik Zetterberg

**Affiliations:** 1 Institute of Neuroscience and Physiology, Department of Psychiatry and Neurochemistry, The Sahlgrenska Academy at the University of Gothenburg, S-405 30, Gothenburg, Sweden; 2 UCL Institute of Neurology, Department of Molecular Neuroscience, University College London, Queen Square, London, WC1N 3BG, UK; 3 Institute of Neuroscience and Physiology, Department of Physiology, The Sahlgrenska Academy at the University of Gothenburg, S-405 30, Gothenburg, Sweden; 4 Department of Clinical Pathology and Cytology, Sahlgrenska University Hospital, S-413 45, Gothenburg, Sweden; 5 Institute of Neuroscience and Physiology, Department of Psychiatry and Neurochemistry, The Sahlgrenska Academy at the University of Gothenburg, S-431 80, Mölndal, Sweden; 6 Institute of Biomedicine, Department of Clinical Chemistry and Transfusion Medicine, The Sahlgrenska Academy at the University of Gothenburg, S-405 30, Gothenburg, Sweden; 7 MRC Centre for Regenerative Medicine, School of Biological Sciences, University of Edinburgh, Edinburgh, EH16 4UU, UK

## Abstract

Amyloid precursor protein (APP) and its cleavage product amyloid β (Aβ) have been thoroughly studied in Alzheimer’s disease. However, APP also appears to be important for neuronal development. Differentiation of induced pluripotent stem cells (iPSCs) towards cortical neurons enables *in vitro* mechanistic studies on human neuronal development. Here, we investigated expression and proteolytic processing of APP during differentiation of human iPSCs towards cortical neurons over a 100-day period. APP expression remained stable during neuronal differentiation, whereas APP processing changed. α-Cleaved soluble APP (sAPPα) was secreted early during differentiation, from neuronal progenitors, while β-cleaved soluble APP (sAPPβ) was first secreted after deep-layer neurons had formed. Short Aβ peptides, including Aβ1-15/16, peaked during the progenitor stage, while processing shifted towards longer peptides, such as Aβ1-40/42, when post-mitotic neurons appeared. This indicates that APP processing is regulated throughout differentiation of cortical neurons and that amyloidogenic APP processing, as reflected by Aβ1-40/42, is associated with mature neuronal phenotypes.

Amyloid precursor protein (APP) is a type I transmembrane protein essential for normal brain development and possibly also for adult brain plasticity^1^. APP and its cleavage product amyloid β (Aβ) have been studied extensively in relation to Alzheimer’s disease (AD)^2^. The Aβ cascade hypothesis, first postulated in 1991^3^, posits that an imbalance in the production and clearance of the aggregation-prone 42 amino acid-long form of Aβ (Aβ1-42) results in deposition of the peptide in senile plaques in the brain parenchyma, which initiates a neurotoxic cascade that ultimately leads to AD dementia^2^.

APP can undergo at least two major proteolytic processing pathways as shown in Fig. 1: (A) the non-amyloidogenic, α-secretase-dependent pathway, in which α-secretase cleaves in the middle of the Aβ sequence precluding formation of full length Aβ. This activity generates a soluble APP fragment (sAPPα) and a membrane-bound C-terminal fragment of APP (αCTF). αCTF can be further cleaved by γ-secretase generating the so-called p3 fragment (Aβ17-40/42); (B) the potentially amyloidogenic pathway in which β-secretase cleaves APP, resulting in secretion of sAPPβ, and a second membrane-bound C-terminal fragment of APP (βCTF). Further cleavage of βCTF by γ-secretase generates several Aβ peptides that start at the first amino acid of the Aβ domain and ends at amino acids 37, 38, 39, 40 or 42^4^. Along with Aβ peptides of different lengths, cleavage of α/βCTFs releases an intracellular domain (AICD) suggested to be involved in gene regulation^5^. Other pathways have also been described lately, including a non-amyloidogenic β-/α-pathway, in which β- and α-secretases act on the same APP molecule resulting in secretion of short soluble Aβ peptides including Aβ1-15/16^6^, and a β-/β-pathway (BACE1/BACE2), resulting in soluble Aβ peptides of approximately 19 to 20 amino acids^7^,^8^ (Fig. 1C). In addition, more than ten N-terminally extended (NTE) Aβ fragments, starting N-terminally of the BACE1 cleavage site and ending at amino acid 15 in the Aβ sequence, have been identified in CSF^9^,^10^. However, whether these NTE fragments contribute to AD pathology remains to be further investigated.

The major α-secretase in the human brain is “A Disintegrin And Metalloproteinase” 10 (ADAM10)^11^, whereas β-site APP-cleaving enzyme 1 (BACE1) is the major β-secretase^12^. γ-Secretase is a multi-protein complex in which the catalytic activity is harboured within the presenilin subunits^13^.

APP is suggested to be involved in several processes in the developing brain, including neuronal differentiation^14^ and promotion of neurite outgrowth and maturation^15^. However, it has been difficult to determine if these effects are due to full length APP or soluble/secreted forms, such as sAPPα. In addition, many endogenous functions of Aβ have been postulated, most of which relate to synaptic homeostasis^16^,^17^,^18^. Low concentrations of Aβ (pM to nM) may have protective effects on neurons and synapses (reviewed in ref. 19). Interestingly, it was recently shown that a short APP fragment, Aβ1-15, augments long term potentiation (LTP) at pM concentrations^20^. Thus, APP and its cleavage products may be important both during neuronal development and in the adult brain.

Research on neurodegenerative diseases, as well as on neuronal development, has been hampered by the lack of relevant human models. The commonly used animal models, overexpressing mutated human APP, do not accurately resemble the human situation and human cell lines are often based on cancer cells and most lack neuronal features. The generation of induced pluripotent stem cells (iPSCs)^21^ and further differentiation of these cells into various types of neurons have opened an avenue of possibilities. In 2012, Shi *et al*. described a method for differentiation of human iPSCs into cortical neurons mimicking the developing steps and time frames seen in human foetal tissue^22^. This not only enables studies of neurological disorders in a clinically relevant cell type, but also allows for investigations of events during the development of human cortical neurons.

Here, we performed a detailed analysis of APP expression and cleavage during differentiation of human iPSCs into cortical neurons, using immunoprecipitation in combination with mass spectrometry as well as immunochemical assays. We investigated (i) if the APP expression pattern varies during neuronal differentiation, (ii) if the pattern of secreted sAPP and Aβ peptides varies during differentiation and (iii) if APP processing and expression can be correlated to important neuronal maturation steps.

## Results

### Expression of cortical maturation markers during differentiation

**Figure 2:**
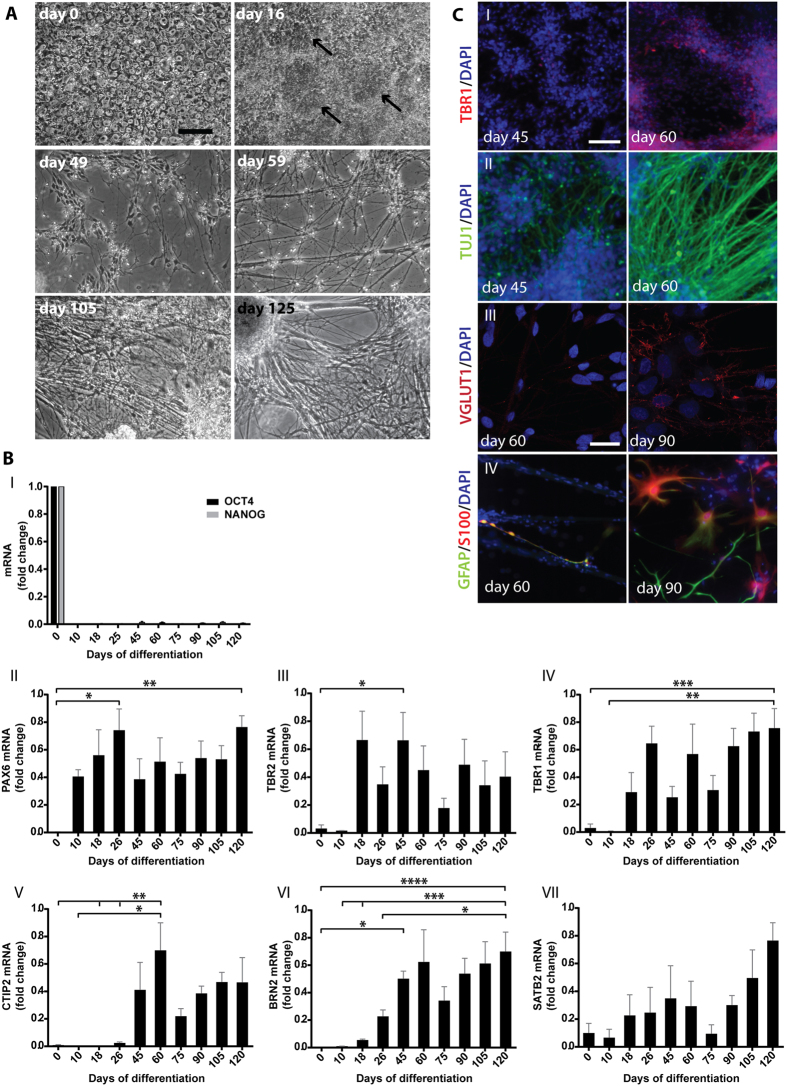
Neuronal maturation during differentiation of human iPSCs to cortical neurons. (**A**) Phase contrast images of cells during differentiation from iPSCs towards cortical neurons show the step-wise expansion and maturation of the neurite network between days 0 and 125. Arrows mark cortical neuronal rosettes formed around day 16. (**B**) qPCR analysis of cortex-specific markers during a total of four separate differentiations of two iPSC lines (A2B and Con1): (I) Expression of the pluripotency markers OCT4 and NANOG are expressed on day 0, but disappear thereafter. (II) PAX6 expression (primary neural progenitor cells) appears on day 10 and remains throughout differentiation. A significant increase in expression compared with day 0 is seen on day 26 and day 120. (III) A clear TBR2 expression (secondary neural progenitor cells) is first observed on day 18 and remains throughout differentiation. A significant increase in expression compared with day 0 is detected on day 45. (IV) TBR1 expression (layer VI neurons) is clearly detected from day 18 and is significantly higher on day 120 compared with days 0–10. (V) CTIP2 (layer V neurons) is steadily expressed from day 45 and throughout differentiation. A significant increase in expression compared with days 0–26 is observed on day 60. (VI) BRN2 (progenitor cells and upper-layer neurons) is expressed from day 18 and increases successively. A significant increase in expression compared with day 0 is seen on day 45 and on day 120 compared with days 0–26. (VII) SATB2 (upper-layer neurons) is expressed at low levels during early stages, with a tendency to increase by the end of differentiation. Bars represent mean+/−SEM, n = 4, except for day 10 where n = 3. *p ≤ 0.05, **p ≤ 0.01, ***p ≤ 0.005, ****p ≤ 0.001. (**C**) Representative images from immunocytochemistry staining of cells during differentiation from human iPSCs towards cortical neurons:(I) TBR1 staining appears and the intensity of (II) TUJ1 staining increases between days 45 and 60. (III) Between days 60 and 90, VGLUT1 staining appears in neurites and intensifies. (IV) Strong GFAP and S100 staining is observed on day 90, but not on day 60. Bars = 50 μM in I-II, 25 μM in III-IV.

iPSCs were differentiated towards cortical neurons using dual SMAD inhibition followed by *in vitro* corticogenesis, according to a protocol by Shi *et al*.^23^. This protocol generates neuronal cells from all cortical layers, as well as astrocytes, after 90–100 days of differentiation. Cortical identity was confirmed by analysis of morphology, as well as mRNA and protein expression of cortical markers throughout the differentiation process, as shown in Fig. 2. Phase contrast images showed the development of neuronal rosettes between days 0 and 16 (Fig. 2A; day 0 and day 16). An extensive outgrowth of neurites could be observed between days 49 and 59 (Fig. 2A; day 49 and day 59) and the network continued to develop until day 105 (Fig. 2A; day 105). Thereafter no further morphological development of the neurite network was observed, but an increasing number of cells were growing in three-dimensional colonies with thick bundles of neurites connecting them (Fig. 2A; day 125). A total of seven separate differentiations of three iPSC lines were performed and used for different analyses. The timing of differentiation differed somewhat between batches, but was not related to iPSC origin.

To monitor the differentiation towards mature cortical neurons in detail, mRNA expression of markers for stem cells, neuronal progenitor cells and mature cortical neurons^21^,^22^,^23^ were analysed from four separate differentiations of two different iPSC lines (Fig. 2B). Expression of the stem cell markers NANOG and OCT4 was detected on day 0, but no expression was detected on day 10 or any other time point during differentiation (Fig. 2B:I). Expression of PAX6, a primary progenitor marker, was detected from day 10 and throughout differentiation (Fig. 2B:II). Compared with day 0, the expression was significantly increased on day 26 and 120. TBR2, a marker of secondary progenitors, was stably expressed from day 18 and the expression was significantly higher on day 45 compared with day 0 (Fig. 2B:III). This indicates that neuronal progenitor cells develop early and remain in the culture throughout differentiation. TBR1, a marker of cortical layer VI neurons, appeared on day 18 and was thereafter expressed throughout differentiation. A significant increase in expression compared with days 0–10 was seen on day 120 (Fig. 2B:IV). Expression of CTIP2, a marker of layer V neurons, was stably detected from day 45 and onwards during differentiation. The expression was significantly higher on day 60, compared with days 0–26 (Fig. 2B:V). Taken together, this indicates a chronological appearance of layer VI and layer V neurons. BRN2 and SATB2 are both markers of upper-layer neurons and their expression increased successively during differentiation. Expression of BRN2 was detected from day 18, but a significant increase compared with day 0 was first observed on day 45 (Fig. 2B:VI). The expression then increased throughout differentiation and on day 120, BRN2 expression was significantly higher than on days 0–26. SATB2 was expressed at low levels during early differentiation, with a tendency to increase successively (Fig. 2B:VII).

Next, immunocytochemistry was performed to investigate expression and localization of neuronal- and glial-specific proteins. A major increase in TBR1 staining was observed between days 45 and 60 (Fig. 2C:I), indicating that the number of deep-layer neurons increased during this period. Similarly, weak staining for TUJ1, a neuron-specific tubulin, was observed on day 45, but increased on day 60 (Fig. 2C:II). Only weak staining for VGLUT1, a protein localized to glutamate-releasing vesicles in cortical neuronal synapses, was detected on day 60 and then mainly localized to nuclei. However, after 90 days of differentiation, strong VGLUT1 staining could be observed throughout the neurite network (Fig. 2C:III), which indicated the presence of functional synapses. Whole-cell patch clamp recordings also confirmed the presence of functional glutamatergic and GABAergic synapses (see Supplementary Fig. S1). Staining for GFAP and S100, markers of astrocytes, was only occasionally observed on day 60, while strong staining was observed on day 90 (Fig. 2C:IV), indicating maturation/appearance of astrocytes at late time points during differentiation.

**Figure 3:**
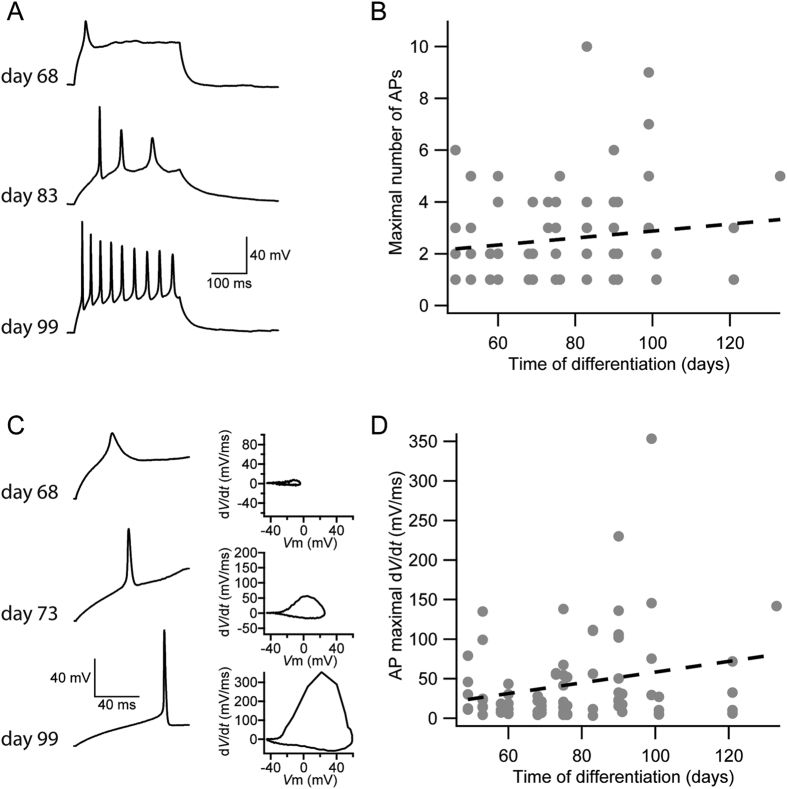
Maturation of action potential properties in neurons derived from human iPSCs. Whole cell current clamp recordings were performed on cells from two separate differentiations of two different iPSC lines (A2B and Con1). (**A**) Example sweeps showing evoked action potentials in response to depolarizing current pulses in cells of three different stages of maturation (measured on days 68, 83 and 99). (**B**) Maximum number of action potentials evoked by the current injections plotted versus time of differentiation (days). A significant correlation between maximal number of action potentials and time of differentiation was observed, r = 0.14, p < 0.001, n = 77. (**C**) Example sweeps of fast and slow rising action potentials evoked by current injections in cells of three different stages of maturation (measured on days 68, 73 and 99). To the right are the corresponding phase plane plots (time derivative of the membrane potential vs. membrane potential) of these action potentials. (**D**) Maximum action potential rate of change plotted versus time of differentiation (days). A significant correlation between rate of action potentials and time of differentiation was observed, r = 0.24, p < 0.001, n = 77. Grey filled circles represent individual neurons. The best fit to the data points in B and D was calculated using linear regression. The significance of the slope was determined using Student’s t-test with n = 2 degrees of freedom.

To further investigate the neuronal maturation throughout differentiation, whole-cell current-clamp recordings were performed during two separate differentiations of two different iPSC lines (example recordings are shown in Fig. 3A). Immature cells (day 68 shown) responded to depolarizing current injection (300 ms) with one action potential, slightly more mature cells (day 83 shown) responded with a few actions potentials with decreasing amplitude, whereas more mature neurons (day 99 shown) responded with a train of action potentials with little decrement of the amplitude. Increasing maturation in the capacity to fire action potentials was examined by plotting the maximal number of action potentials evoked by the depolarizing pulse against time of differentiation (Fig. 3B). Although there was a statistically significant positive correlation (r = 0.14, p < 0.001, n = 77) between maximal number of action potentials and time of differentiation, it is important to note that time of differentiation only explains less than two percent (0.14 × 0.14) of the variability in the material.

Additionally, the maximal rate of change (d*V*/d*t*) of the first evoked action potential^24^ was calculated by plotting the time derivative of the membrane potential during the action potential against membrane potential. Figure 3C illustrates examples of action potentials from three different cells, of which one displayed a slow rate of change (day 68; 8.4 mV/ms, Fig. 3C, upper panel), one a moderate rate of change (day 73; 56.9 mV/ms, Fig. 3C, middle panel) and one a fast rate of change (day 99; 353.4 mV/ms, Fig. 3C, lower panel). There was also statistically significant positive correlation (r = 0.24, p < 0.001, n = 77) between maximal number of action potentials and time of differentiation (Fig. 3D), although it is important to note that time of differentiation only explains about six percent (0.24 × 0.24) of the variability in the material. Taken together, these data are consistent with a general neuronal maturation from day 50 to day 120 of differentiation. However, the cultures are heterogeneous containing both immature progenitor cells and mature neurons at all examined time points.

### APP is expressed throughout cortical neuronal differentiation

**Figure 4:**
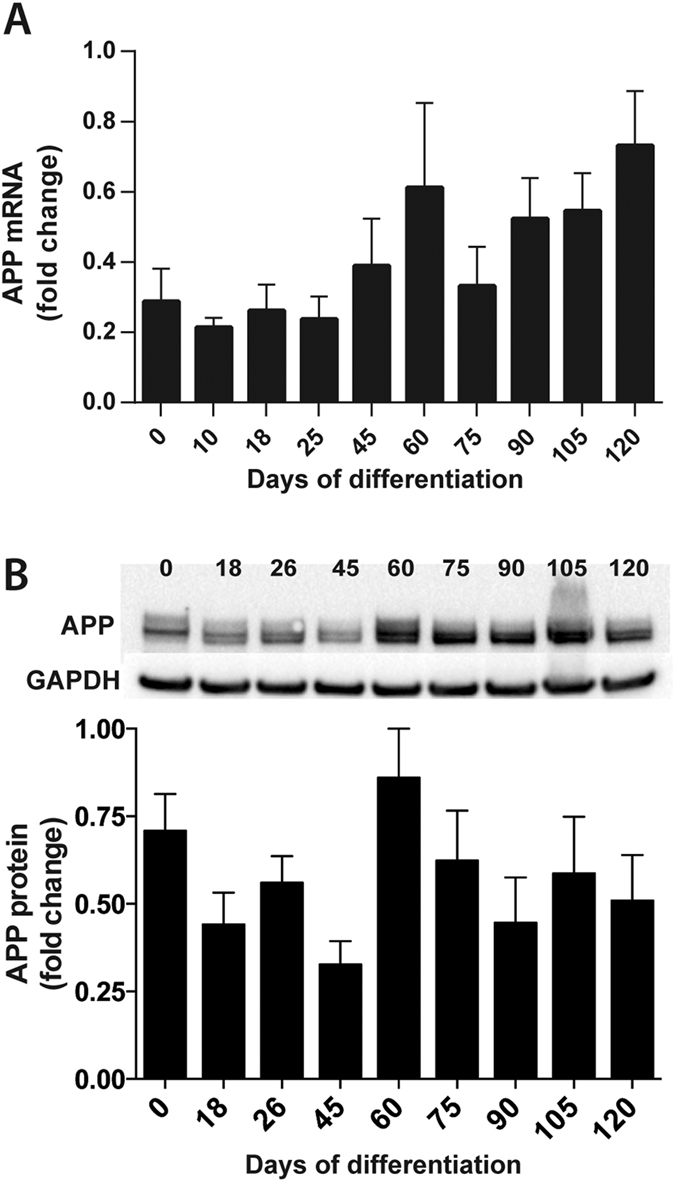
Intracellular levels of APP mRNA and protein during neuronal differentiation. Cells from four separate differentiations of two iPSC lines (A2B and Con1) were collected at different time points during differentiation of human iPSCs towards cortical neurons and analysed for APP mRNA and protein expression. (**A**) APP mRNA, as measured with qPCR, is expressed from day 0 (undifferentiated iPSCs). A tendency towards increased expression throughout differentiation is observed. Each sample is calibrated to the average gene expression of *RPL27*/*RPL30*/*HPRT1* and related to the sample with highest APP expression in each experiment, set to one. Bars represent mean +/−SEM, n = 4 (except for day 10, where n = 3). (**B**) Intracellular APP protein, as measured with Western blot, shows that APP is expressed from day 0 (undifferentiated iPSCs). Expression remained relatively unchanged during differentiation. One representative blot out of four is shown. Each sample was correlated to GAPDH and related to the sample with highest expression in each experiment, set to one. Bars represent mean +/−SEM, n = 4 (except for day 120, where n = 2).

Since APP is suggested to be involved in neuronal development and maturation^14^, both APP mRNA and APP protein expression was investigated during four separate differentiations of two different iPSC lines, shown in Fig. 4. Quantitative PCR performed on cells collected at various time points during differentiation showed that APP mRNA (Fig. 4A) was stably expressed from day 0 (undifferentiated iPSCs). Though no significant changes in APP mRNA expression were detected, a trend towards increased expression of APP mRNA could be noticed from days 45–60 until the end of differentiation. Western blot analysis of intracellular APP protein expression (Fig. 4B) also showed expression of APP from day 0, which remained relatively unchanged during differentiation.

### The concentrations of secreted sAPP and Aβx-38/40/42 change during cortical neuronal differentiation

**Figure 5:**
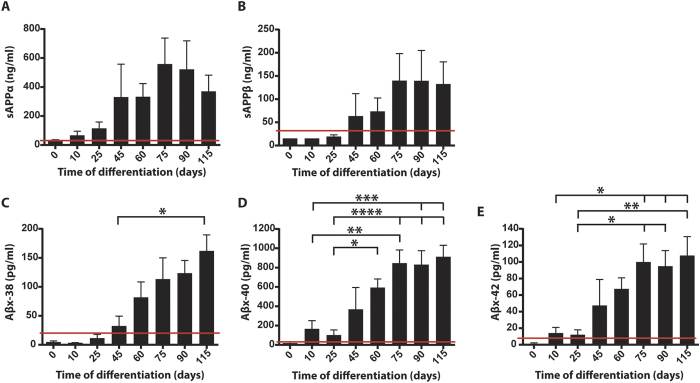
Secreted levels of sAPPα/β and Aβx-38/40/42 increase with the formation of cortical neurons. Conditioned cell culture media was collected throughout five separate differentiations of three iPSC lines (A2B, BJ1a and Con1) towards cortical neurons and secreted sAPP and Aβ was quantified using immunochemical methods. (**A**) Secretion of sAPPα is detected from day 10 of differentiation and tends to peak on day 75. (**B**) Secretion of sAPPβ is detected from day 45 and remains stable from day 75. (**C**) Secretion of Aβx-38 is detected from day 45 and increases thereafter. A significant increase in secretion is detected on day 115 compared with day 45. (**D-E**) Secretion of Aβx-40 and Aβx-42 is detected at low levels from day 10 of differentiation, but increases dramatically between days 45 and 75. (**D**) On days 75–115, secretion of Aβx-40 was significantly higher compared with day 10, and on days 60–115 compared with day 25. (**E**) On days 75–115, secretion of Aβx-42 was significantly higher compared with days 10–25. Red lines indicate the lower limit of detection for all five experiments. Bars represent mean +/−SEM, n = 5. *p ≤ 0.05, **p ≤ 0.01, ***p ≤ 0.005, ****p ≤ 0.001. Samples below detection limit in each experiment are plotted as detection limit value/2.

Different cleavage fragments of APP, both Aβ peptides and the soluble forms of APP (sAPPα and sAPPβ), are suggested to be involved in neurogenesis and synaptic development (reviewed in ref. 25). Therefore, cleavage of APP during neuronal differentiation was investigated by quantification of sAPPα/β and Aβx-38/40/42 secreted into cell culture media, using immunochemical methods. The media from five separate differentiations of three different iPSC lines was analysed. sAPPα and sAPPβ showed different secretion patterns (Fig. 5A,B). sAPPα was detected in the culture media from day 10 of differentiation, increased steadily until day 75 and decreased thereafter (Fig. 5A). In comparison, sAPPβ was not detected until day 45 and the levels remained stable after day 75 (Fig. 5B). Secretion of Aβx-38 was detected from day 45 of differentiation and increased steadily thereafter (Fig. 5C). A significant increase in secretion was seen on day 115, compared with day 45. Secretion of both Aβx-40 and Aβx-42 was detected at low levels from day 10 and increased from day 45 throughout differentiation. On days 75–115, secretion of Aβx-40 was significantly higher as compared to day 10 and on days 60–115 as compared to day 25 (Fig. 5D). On days 75–115, secretion of Aβx-42 was significantly higher as compared to days 10–25 (Fig. 5E). As in human cerebrospinal fluid (CSF)^26^, Aβx-40 concentrations were almost ten-fold to Aβx-38 and Aβx-42 concentrations (Fig. 5C–E).

### Shift in secreted Aβ peptides during cortical neuronal differentiation

**Figure 6:**
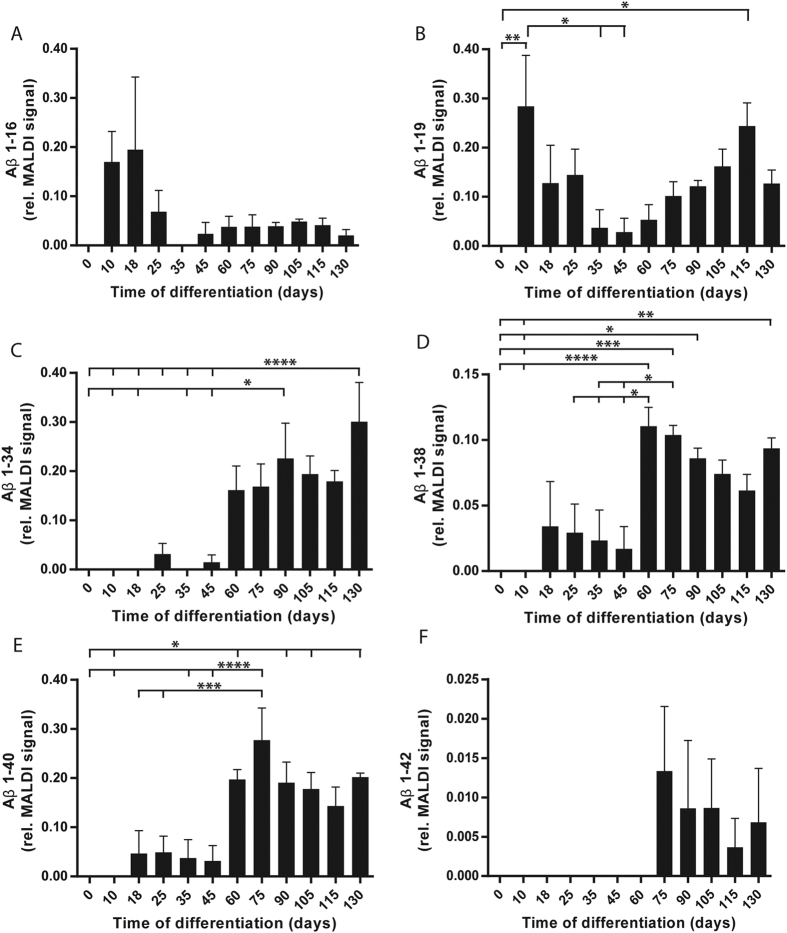
Secretion of different Aβ peptides shifts during neuronal differentiation. Conditioned cell culture media was collected throughout four separate differentiations of two iPSC lines (A2B and Con1) towards cortical neurons, and secreted Aβ peptides were detected using IP-MS. (**A**) Aβ1-16 is detected on day 10 of differentiation and the maximum relative levels are reached on day 18 to thereafter remain at a low level. (**B**) Aβ1-19 is detected at the highest relative level on day 10 of differentiation, significantly higher compared with day 0. The relative levels thereafter decrease, to be significantly lower on days 35–45 as compared to day 10. The relative levels thereafter increase, and on day 115, the levels are again significantly higher as compared to day 0. (**C**) Secretion of Aβ1-34 is first detected on day 25 and an increase in relative levels is seen from day 60 until the end of the differentiation protocol. Secreted levels on day 90 are significantly higher compared with days 0–18 and 35–45 and significantly higher on day 130 compared with days 0–45. (**D**) Aβ1-38 is detected from day 18 and the relative levels increase on day 60, with significantly higher levels than on days 0–10 and 25–45. On day 75, the levels are significantly higher than on days 0–10 and 35–45 and are significantly higher on days 90 and 130 compared with days 0–10. (**E**) Aβ1-40 is detected from day 18, with significantly higher levels on day 60, 90, 105 and 130 compared with days 0–10 and on day 75 compared with days 0–45. (**F**) Secretion of Aβ1-42 is not detected until day 75, with a tendency towards decreasing relative levels thereafter. Bars represent mean +/−SEM of the area under curve (AUC) for each respective Aβ peptide related to the total AUC of all identified Aβ peptides in the same sample, n = 4. *p ≤ 0.05, **p ≤ 0.01, ***p ≤ 0.005, ****p ≤ 0.001.

To examine the relative secretion of a wider range of Aβ peptides into cell culture media during neuronal development, media collected throughout four separate differentiations of two different iPSC lines were analysed by immunoprecipitation mass spectrometry (IP-MS). This method detects Aβ peptides of different lengths, from 14 to 42 amino acids long, representing both amyloidogenic and non-amyloidogenic APP-processing pathways^27^. Altogether, 12 Aβ peptides were reproducibly detected and used to calculate the Aβ peptide ratios (relative levels) (see Supplementary Fig. S2). Six of these, representing relevant APP processing pathways during differentiation, are displayed in Fig. 6. No Aβ peptides were detected in the culture media from undifferentiated iPSCs (day 0). Secretion of Aβ1-16 (Fig. 6A) was detected already after 10 days of differentiation, peaked after 18 days and declined thereafter to low, stable levels. Aβ1-19 (Fig. 6B) displayed the highest relative levels after 10 days, which was significantly higher as compared to day 0. Thereafter, the levels decreased and were significantly lower on days 35–45 than on day 10. Towards the end of differentiation, a gradual increase was detected and on day 115 the levels were again significantly higher than on day 0. Low relative levels of Aβ1-34 (Fig. 6C) were detected on days 25 and 45, while increased relative levels were detected from day 60, with a significant increase on day 90 compared with days 0–18 and 35–45, and on day 130 compared with days 0–45. The longer Aβ peptides Aβ1-38 and Aβ1-40 (Fig. 6D,E), displayed low relative levels from day 18, but increased dramatically on day 60. Relative levels of Aβ1-38 were significantly higher on day 60 compared with days 0–10 and 25–45, on day 75 compared with days 0–10 and 35–45 and on days 90 and 130 compared with days 0–10 (Fig. 6D). Relative levels of Aβ1-40 were significantly higher on days 60, 90, 105 and 130 compared with days 0–10, and on day 75 compared with days 0–45 (Fig. 6E). Secretion of Aβ1-42 was not detected until day 75 and thereafter the relative levels tended to decline gradually (Fig. 6F).

### Increasing BACE1 mRNA expression during cortical neuronal differentiation

**Figure 7:**
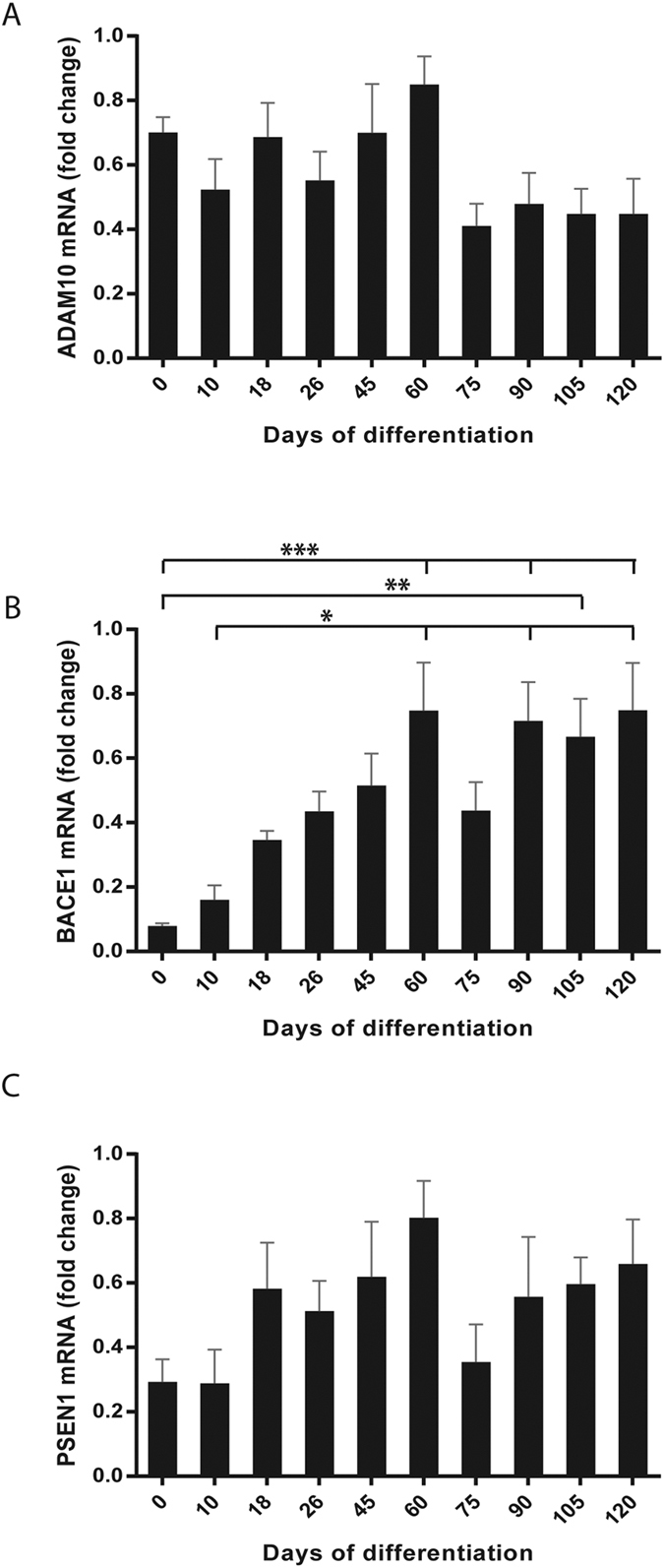
Intracellular mRNA levels of APP-cleaving secretases during neuronal differentiation. Cells from four separate differentiations of two iPSC lines (A2B and Con1) were collected at different time points during differentiation of human iPSCs towards cortical neurons and analysed for *ADAM10*, *BACE1* and *PSEN1* mRNA expression. (**A**) *ADAM10* mRNA, as measured with qPCR, is expressed from day 0 (undifferentiated iPSCs). The expression levels remain relatively stable throughout differentiation but with a tendency towards stably lower levels after day 60. (**B**) *BACE1* mRNA, as measured with qPCR, is expressed at low levels from day 0 (undifferentiated iPSCs). The levels increase with time of differentiation until day 60, to thereafter remain stable. The relative levels on day 60 and days 90–120 are significantly higher as compared to day 0, and on days 60, 90 and 120 significantly higher as compared to day 10. (**C**) *PSEN1* mRNA, as measured with qPCR, is expressed from day 0 (undifferentiated iPSCs) with a tendency to increased levels between days 10 and 60. Each sample is calibrated to the average gene expression of *RPL27*/*RPL30*/*HPRT1* and related to the sample with highest mRNA expression in each experiment, set to one. Bars represent mean +/−SEM, n = 4 (except for day 10, where n = 3). *p ≤ 0.05, **p ≤ 0.01, ***p ≤ 0.005.

To investigate if the difference in APP processing during neuronal differentiation could be due to regulated expression of the APP-cleaving secretases, quantitative PCR was performed on cells collected at various time points during four separate differentiations of two different iPSC lines. *ADAM10* mRNA was stably expressed throughout differentiation, with a tendency to decreased levels after day 60 (Fig. 7A). *BACE1* mRNA expression was relatively low on day 0 (undifferentiated iPSCs) and increased gradually until day 60 (Fig. 7B), to be significantly higher on days 60 and 90–120 as compared to day 0. The levels were also significantly higher on days 60, 90 and 120 as compared to day 10. *PSEN1* (the active subunit of the γ-secretase complex) mRNA expression was detected from day 0 and a tendency towards increased expression was seen between days 10 and 60 (Fig. 7C).

## Discussion

APP may play important roles during neuronal development as well as in the adult brain^28^. Here, we performed a detailed analysis of the expression and processing of APP during differentiation of human iPSCs towards cortical neurons. The results show that the proteolytic processing of APP during neuronal differentiation from iPSCs can be separated into three stages: (i) the undifferentiated stage when APP is expressed but no APP cleavage occurs, (ii) the neuronal progenitor stage, when APP is processed primarily along non-amyloidogenic pathways and (iii) the neuronal stage when β-/γ-secretase processing of APP leads to secretion of longer, potentially amyloidogenic Aβ peptides.

Our first observation was that although APP is expressed on both mRNA and protein levels in human iPSCs, neither sAPP nor Aβ was secreted into the cell culture media. There could be several reasons for this. Firstly, expression of the secretases responsible for APP cleavage could be low in iPSCs. However, an earlier study showed that mRNA of all relevant components of α-, β- and γ-secretase are expressed in iPSCs^29^. In line with this, we found that *ADAM10*, *BACE1* and *PSEN1* were all expressed at mRNA level during the undifferentiated iPSC stage. Another reason could be that the necessary co-localization of APP and secretases is lacking in iPSCs^30^. Alternatively, differences in APP splice variants or post translational modifications could potentially be relevant for the lack of processing that we detect at this stage.

Next, we found that already after ten days of neuronal differentiation, around the same time as PAX6-expressing neuronal progenitor cells appear in the cultures, the cells enter a second stage where APP is processed and secreted. This is probably not due to a change in APP expression, as no correlation between APP expression and APP processing was seen throughout differentiation. At this stage, most APP is cleaved along the non-amyloidogenic pathway, reflected by secretion of sAPPα (a result of α-secretase cleavage). Secretion of sAPPα is detected in the culture media from day 10, in line with an earlier study carried out in different iPSC lines using another differentiation protocol^31^. Additionally, the short Aβ peptides Aβ1-16 and Aβ1-19 are detected early during the progenitor stage and peak in the media between days 10 and 18 of differentiation. Aβ1-16 is generated through a concerted β- and α-secretase pathway^32^. It is presently unclear which enzymes are responsible for generation of Aβ1-19. Cleavage of APP between amino acids 19 and 20 has been suggested to depend on a second β-secretase (BACE2)^6^,^8^, although another study suggests that γ-secretase cleavage has to occur prior to BACE2 cleavage of APP after amino acid 19^33^.

The presence of short Aβ peptides, and the absence of longer ones, in neuronal progenitor cells indicate low rate of γ-secretase cleavage of APP at this stage. Since expression of *PSEN1* mRNA was detected throughout differentiation, this could instead be regulated by APP/secretase co-localization. α-Cleavage takes place at the plasma membrane by a number of secretases and sheddases^34^. Our data show that generation of short Aβ peptides can occur before the establishment of mature synapses. This pathway may thus also take place at the plasma membrane without the involvement of synaptic vesicle recycling that has been suggested important for the classical β- and γ-secretase-mediated generation of longer Aβ forms^35^. Alternatively it is possible that NOTCH, one of many γ-secretase substrates and a protein highly involved in neuronal progenitor development and regulation^36^, competes with cleavage of the CTFs of APP that are left in the membrane following α- and/or β-secretase cleavage of APP^37^. Release of sAPPα and short Aβ peptides from APP indicates that both α- and β-secretases are active during this stage. However, the lack of detectable sAPPβ in the cell media is inconsistent with this simplistic model. As the sAPPα and sAPPβ assays we employed have very similar lower limits of detection, the selective detection of sAPPα most likely reflects a real difference in the secreted APP profile. Detection of Aβ peptides, and not sAPPβ could instead be due to differences in sensitivity between the Aβ and sAPP assays (more than 1000-fold difference), or that sAPPβ released during APP processing is re-internalized and degraded. Alternatively, additional proteases cleave around the β-secretase site so that we do not detect a parallel increase in sAPPβ when the short Aβ peptides are produced.

Finally, we found that during a third stage of differentiation, when post-mitotic neurons are formed, a shift occurs in APP processing, favouring longer Aβ peptides generated by the β/γ pathway. After days 18–25 of differentiation, IP-MS measurements show a decrease in Aβ1-16/19 secretion and simultaneously a detection of Aβ1-34/38/40, indicating a shift in favour of β/γ- over β/α-secretase cleavage. Generation of Aβ1-34 is believed to result from dual β-secretase cleavage at Aβ amino acid 34, although a preceding γ-secretase cleavage seems to be necessary^38^. The steady increase in longer peptides at later stages therefore indicates an increasing activity of both β- and γ-secretase over time. Interestingly, while *ADAM10* was expressed at relatively high levels in iPSCs and immature neurons, with a tendency to drop to stably lower levels after 60 days of differentiation, *BACE1* mRNA levels instead increased successively up to day 60 of differentiation and thereafter the levels plateaued. *PSEN1* showed a similar increase until day 60, although the change failed to reach statistical significance. Secretion of Aβ1-42 is not detected with IP-MS until day 75 of differentiation, but the more sensitive and quantitative immunochemical assay shows a marked increase of Aβx-38/40/42 levels in the media along with increased sAPPβ secretion on days 45 to 60. Immunocytochemistry images show that TBR1-positive cells appear around day 45, indicating formation of deep-layer neurons, and increase in number until day 60 as more mature neurons with extensive neurite network develop. As an indication of synaptic maturation, an increased VGLUT1 staining in neurites was observed between days 60 and 90. During this period we also demonstrated the presence of functional glutamatergic and GABAergic synapses and an increasing capability of the neurons to generate trains of action potentials. This period was also associated with an increase in S100/GFAP-positive astrocytes, which are critical for the generation and maturation of functional synapses^39^.

Generation of longer Aβ peptides is believed to require endocytosis of APP^35^ and it has also been suggested that synaptic vesicles could be a part of this process^40^,^41^. It can therefore be speculated that the amyloidogenic β/γ-pathway only can take place when neurons have developed an elaborate neurite network with functional synapses.

It should be noted that the data presented here includes three different iPSC lines from two different cellular origins (fibroblast and chondrocyte). This indicates that the pattern of secretion is independent of iPSC line and type of cellular origin, despite relatively large variation in the data. The variation in both mRNA expression of neuronal maturation markers and electrophysiological properties could be due to inherent differences in iPSC lines or slight differences in the timing of the differentiation between batches. Furthermore, our results regarding secretion of sAPP and Aβ1-40/42 are in line with previously published results using another method of neuronal differentiation^31^. This cell model thus demonstrates a developmental secretion pattern of sAPP and Aβ that could be used for further studies in elucidating the biological function of APP and its cleavage products.

Taken together, the finding that APP is cleaved in human neuronal progenitors and neurons, but not in human iPSCs, indicates that this type of directed APP cleavage is specific for cells of neuronal linage. In addition, the shift towards β/γ-cleavage of APP that takes place when neuronal progenitor cells mature into functional neurons indicates that amyloidogenic Aβ formation is linked to mature neuronal functions.

## Methods

### Cell culture

Three different iPSC lines were used in this study, femoral condyle chondrocyte-derived iPSCs (A2B)^42^, fibroblast-derived iPSCs (Con1)^43^ and in-house reprogrammed fibroblast-derived iPSCs (BJ1a). Human BJ fibroblasts (Stemgent) were cultured in DMEM/F12 (LifeTechnologies) with 10% fetal bovine serum (SigmaAldrich) in a humidified atmosphere at 37 °C and 7% CO_2_. mRNA reprogramming was conducted using 6-factor mRNA mix from Allele Biotechnology, where wild type OCT4 is replaced by M3O, an engineered OCT4-protein containing the DNA binding moiety of OCT4 and the transactivating domain of MyoD^44^,^45^,^46^. The entire reprogramming cycle was conducted in Pluriton Reprogramming Medium (Stemgent) under hypoxic conditions in a humidified atmosphere at 37 °C, 5% CO_2_ and 5% O_2_. Briefly, BJ fibroblasts (100 k/cm^2^) were seeded in 6-well tissue culture plates coated with CELLstart CTS (LifeTechnologies) according to the manufacturer’s instructions. mRNA transfections were conducted daily for 9 consecutive days using Stemfect (Stemgent) with daily addition of 200 ng/ml B18R interferon inhibitor (eBioscience). B18R supplementation was discontinued the day after the final transfection. After additional ten days of daily medium changes, clonal iPSC lines were established by manually picking hESC-like colonies to LN521-coated plates (BioLamina) in NutriStem medium (Biological Industries). Clones were expanded by single cell enzymatic passage using TrypLE Select (Life Technologies) on LN521 in NutriStem. A2B and BJ1a iPSCs were maintained in DEF-CS (Cellartis by Takara-Bio Europe). Con1 iPSCs were cultured on Geltrex-coated plates in complete Essential-8 medium (both from Life Technologies) according to manufacturer’s instructions. All cell cultures were kept in a humidified atmosphere at 5% CO_2_ and 37 °C.

### Differentiation to cortical human neurons

Human iPSCs were differentiated towards cortical neurons according to Shi *et al*.^23^. In brief, when the cells attained 100% confluency, neural induction was initiated by changing to neural maintenance media (NMM; a 1:1 mixture of DMEM/F12 and neurobasal media supplemented with: 1 × N2, 1 × B27, 50 μM 2-mercaptoethanol, 0.5 × non-essential amino acids, 100 μM L-Glutamine (all from Life Technologies), 2500 U/ml Penicillin/Streptomycin (GE Healthcare), 10 μg/ml insulin and 0.5 mM sodium pyruvate (both from Sigma-Aldrich)) further supplemented with 500 ng/ml mouse Noggin-CF chimera (R&D Systems) and 10 μM SB431542 (Stemgent). Cells were maintained in this medium for nine to eleven days until attaining the characteristic neuroepithelial cell morphology. The neuroepithelial sheet was then dissociated with dispase (10 mg/ml; Life Technologies) and re-plated in NMM supplemented with 20 ng/ml FGF2 (Peprotech) on laminin-coated plates (1 μg/cm^2^; Sigma-Aldrich) for 3–4 days. To remove cells differentiation towards unwanted cell types, cell colonies were further passaged with dispase before day 25. When substantial neurogenesis had occurred, the cells were dissociated with StemPro Accutase (Life technologies) to obtain single cells. The cells were further passaged with StemPro Accutase before the final seed out around day 35. Cells were then re-plated onto poly-_L_-ornithine (0.01%; Sigma-Aldrich) and laminin-coated plates in NMM, and maintained for further two to four months with change of media every second day.

### Total RNA extraction and cDNA synthesis

The cells were lysed directly in the well by addition of 600 μl Buffer RLT supplemented with 4 mM dithiothreitol (Sigma-Aldrich) after a wash with Dulbecco’s Phosphate-Buffered Saline (DPBS, Life Technologies). Total RNA was extracted and purified on a Qiacube robotic work station (Qiagen), using the RNeasy Mini protocol according to manufacturer’s instructions. Total RNA concentration was measured on a NanoDrop 2000/2000c spectrophotometer (ThermoScientific) and diluted in RNase-free water to a final concentration of 25 ng/μl. cDNA was synthesised from 250 ng of total RNA using a High Capacity cDNA kit with RNase inhibitor (Applied Biosystems) in a total reaction volume of 20 μl and converted in a single-cycle reaction on a 2720 Thermal Cycler (Applied Biosystems): 25 °C for 10 min, 37 °C for 120 min and 85 °C for 5 min.

### Quantitative PCR

Quantitative PCR was performed using inventoried TaqMan Gene Expression Assays with FAM reporter dye in TaqMan Universal PCR Master Mix with UNG according to protocol, but in a total reaction volume of 25 μl. qPCR reactions were carried out on Micro-Amp 96-well optical microtitre plates on a 7900HT Fast QPCR System (Applied Biosystems), using standard settings for Standard Curve qPCR. TaqMan Gene Expression Assays for the following genes were used: Nanog homeobox (*NANOG*: Hs04399610_g1); POU class 5 homeobox 1 (*POU5F1*/OCT4: Hs01895061_u1); paired box 6 (*PAX6*: Hs00242217_m1); eomesodermin (*EOMES*/TBR2: Hs00172872_m1); T-box, brain, 1 (*TBR1*: Hs00232429_m1); B-cell CLL/lymphoma 11B (*BCL11B*/CTIP2: Hs01102259_m1); POU class 3 homeobox 2 (*POU3F2*/BRN2: Hs00271595_s1); SATB homeobox 2 (*SATB2*: Hs00392652_m1); ADAM metallopeptidase domain 10 (*ADAM10*: Hs00153853_m1); beta-site APP-cleaving enzyme 1 (*BACE1*: Hs01121195_m1); presenilin 1 (*PSEN1*: Hs00997789_m1); ribosomal protein L27 (*RPL27*: Hs03044961_g1); ribosomal protein L30 (*RPL30*: Hs00265497_m1); hypoxanthine phosphoribosyltransferase 1 (*HPRT1*: Hs02800695_m1). 2.5 ng cDNA was used in the PCR and all samples were run in duplicates. PCR results were analysed with the SDS 2.3 software (Applied Biosystems) and the relative quantity was determined using the ΔΔC_T_ method^47^, with the sample with highest expression as calibrator and average C_T_:s of RPL27, RPL30 and HPRT1 as endogenous reference.

### Immunocytochemistry

For immunocytochemistry (ICC), cells were differentiated on poly-_L_-ornithine- and laminin-coated chamber slides (Ibidi). The cells were fixed in Histofix (Histolab AB) for 20 min at room temperature and stained as described previously^42^. Briefly, cells were permeabilized using 0.1% Triton-X100 in DPBS, and thereafter blocked in block buffer (0.1 M glycine, 2% BSA, 0.1% Triton-X100 in PBS). Primary antibodies, TBR2 1:200 (Abcam ab23345), TUJ1 1:1000 (Abcam ab14545), TBR1 1:300 (Abcam ab31940), GFAP 1:1000 (Abcam ab4674), S100 1:400 (Dako Z0311) and VGLUT1 1:750 (Synaptic Systems 135303), were diluted in block buffer and incubated at 4 °C overnight. After washing and re-blocking, samples were incubated with alexa488- and alexa647-conjugated secondary antibodies (Life Technologies) for 2 hours at room temperature and thereafter mounted using Ibidi mounting media (Ibidi).

The samples were analysed using a Nikon EclipseTi inverted fluorescent microscope with 10–20× objectives, and images were captured using the DU-897 Andor camera and the Nis Elements software (Nikon). Alternatively, samples were analysed using a Zeiss LSM700 inverted confocal microscope with 40–63× objectives and the ZEN2000 software (Zeiss). Image analysis was performed using ImageJ (NIH).

### Electrophysiology recordings

For electrophysiological experiments, cells were differentiated in Ibidi μ-dishes (Ibidi). The μ-dishes were mounted under a microscope (Nikon E600FN) where the cells were perfused (2–3 ml/min) with artificial CSF (ASCF) containing (in mM): 1.25 NaH_2_PO_4_, 124 NaCl, 26 NaHCO_3_, 3 KCl,2 MgCl_2_, 1 CaCl_2_ and 10 D-glucose. The ACSF was continuously bubbled with gas containing 95% O_2_ and 5% CO_2_. Patch-clamp recordings were performed on cells visually identified using infrared differential interference contrast video microscopy. Data were acquired with a patch clamp amplifier (EPC-10, Heka Elektronik) at a sampling frequency of 20 kHz and filtered at 2.9 kHz. Patch pipettes (3–7 MΩ) were pulled using a horizontal Flaming/Brown (P-97, Sutter Instrument Company) or a laser-based (P-2000, Sutter Instrument Company) micropipette puller. The pipette solution contained (in mM): 127 K-gluconate, 8 KCl, 10 Hepes, 15 phosphocreatine, 4 Mg-ATP and 0.3 Na-GTP (pH 7.2, 295 mosmol Kg^−1^). To record action potentials cells were current clamped at −70 mV and a series of 15 hyperpolarizing and depolarizing current injections (−20–50 pA and −20–100 pA, 300 ms duration) were applied. Analyses of action potentials were done using Igor Pro (WaveMetrics Inc,). To record spontaneous excitatory postsynaptic currents (EPSCs) and inhibitory postsynaptic currents (IPSCs) the cells were voltage clamped at −70 mv and 0 mV, respectively. Analyses of spontaneous EPSCs/IPSCs were performed using the Mini Analysis Program (Synaptosoft Inc,). Picrotoxin (PTX) (Sigma-Aldrich) was used to block GABA_A_ mediated currents and 6-cyano-7-nitroquinoxaline-2,3-dione (CNQX) (Tocris) was used to block AMPA-mediated currents. Series resistance was monitored using a 10 mV hyperpolarizing pulse and was not allowed to exceed 30 MΩ.

### Western blot

For Western blot analysis, cells were lysed in RIPA buffer (20 mM Tris-HCl, pH 7.5, 150 mM NaCl, 1 mM EDTA, 1% Triton X-100, 0.5% deoxycholate 0.1% SDS) supplemented with MiniComplete protease inhibitor cocktail (Roche), sonicated for 10 min and incubated on ice for 20 min. Thereafter, the samples were centrifuged at 4000 g, 5 min at 4 °C and the supernatant were transferred to fresh tubes and stored at −80 °C until further analysis. Protein determination was performed using the Pierce BCA protein assay kit (ThermoFisher Scientific). Loading buffer was added to equal amounts of protein, and samples were loaded onto a 4–12% Bis-tris gel and transferred using MES buffer (all from Life technologies). Proteins were blotted onto a 0.2 μM nitrocellulose membrane (GE Healthcare) using semi-dry technique. Membranes were blocked in 5% non-fat dry milk (BioRad laboratories) and incubated over night at 4 °C with primary antibody 6E10 (Signet Laboratories). After washing, membranes were incubated with HRP-conjugated anti-mouse secondary antibody (Cell Signaling Technologies) for 1 hour at room temperature. For protein detection, SuperSignal West Dura Extended Duration Substrate (ThermoFisher Scientific) was used and bands were visualised using ChemiDoc XRS+ (BioRad laboratories). Membranes were thereafter stripped using Restore stripping buffer (ThermoFisher Scientific) and re-probed with an HRP-conjugated glyceraldehyde-3-phosphate dehydrogenase (GAPDH) antibody (Novus Biologicals). Band intensities were calculated using ImageJ (NIH) and APP intensity was correlated to GAPDH. All samples were thereafter related to the sample with highest ratio in each run and presented as median +/−SEM.

### Cell culture media sample collection

Conditioned cell culture media was collected after 48 hours of incubation and centrifuged at 360 g for five min (to remove cell debris) before the supernatant was transferred to new tubes and stored at −80 °C.

### Immunochemical quantification of sAPPα, sAPPβ and Aβ peptides

Cell-conditioned media concentrations of sAPPα and sAPPβ were determined using the MSD sAPPα/sAPPβ Multiplex Assay, as described by the manufacturer (Meso Scale Discovery). This assay employs the 6E10 antibody to capture sAPPα and a neoepitope-specific antibody to capture sAPPβ. Both isoforms are detected by SULFO-TAG-labelled anti-APP antibody p2-1. Media concentrations of Aβx-38/40/42 were measured using the MSD Human (6E10) Abeta Triplex Assay as described by the manufacturer (Meso Scale Discovery). This assay employs C-terminally specific antibodies to capture Aβx-38/40/42, respectively, and the 6E10 antibody in combination with a SULFO-TAG-labelled anti-6E10 to quantify the peptides. The limit of detection was set to the value of the lowest standard point.

### Immunoprecipitation and mass spectrometry

Cell-conditioned media collected throughout differentiation was investigated for Aβ peptides using IP-MS as described previously^27^. Briefly, 4 μg of the anti-Aβ antibodies 6E10 and 4G8 (Signet Laboratories) was separately added to 50 μl each of magnetic Dynabeads M-280 Sheep Anti-Mouse IgG (Invitrogen) and used for immunoprecipitation (IP). Mass spectrometry measurements were performed using a Bruker Daltonics UltraFleXtreme matrix-assisted-laser-desorption/ionization time-of-flight/time-of-flight (MALDI TOF/TOF) instrument or a Bruker Daltonics AutoFlex MALDI TOF (Bruker Daltonics). All samples were analysed in duplicate. The area under curve (AUC) of each peak was normalized against the AUC sum for all Aβ peaks in the spectrum (relative MALDI signal).

### Statistical Analyses

All statistical analyses were performed using the IBM SPSS Statistics 21 software (IBM). Mean values were compared using one-way analysis of variance (ANOVA) followed by Tukey’s post hoc analysis. P-values < 0.05 were considered statistically significant.

## Additional Information

**How to cite this article**: Bergström, P. *et al*. Amyloid precursor protein expression and processing are differentially regulated during cortical neuron differentiation. *Sci. Rep.* **6**, 29200; doi: 10.1038/srep29200 (2016).

## Supplementary Material

Supplementary Information

## Figures and Tables

**Figure 1:**
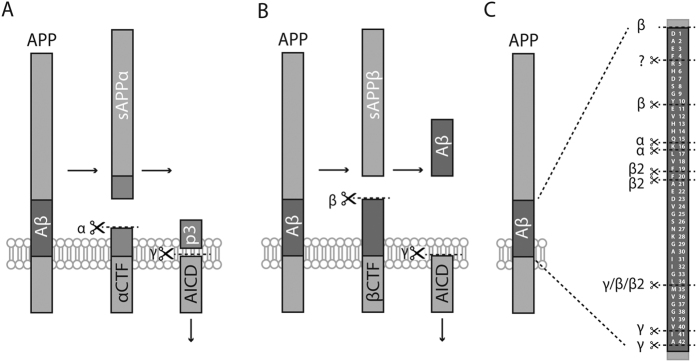
Schematic illustration of the cleavage sites for α- β- and γ-secretase on amyloid precursor protein (APP). (**A**) α-Secretase cleavage of APP around Aβ amino acids 15–16, results in release of soluble APPα (sAPPα), leaving a C-terminal fragment (αCTF) in the membrane available for further processing. Cleavage of αCTF by γ-secretase results in release of the p3 peptide and the amyloid intracellular domain (AICD). (**B**) β-Secretase cleavage of APP at Aβ amino acid 1 results in release of sAPPβ, leaving a C-terminal fragment (βCTF) in the membrane available for further processing. Further cleavage of βCTF by γ-secretase results in release of longer Aβ peptides, generally 38–42 amino acids in lengths. (**C**) In addition to the 38–42 amino-acid long Aβ peptides, other peptides can be generated through alternative cleavage sites for α- and β-secretase. β-Secretase also cleaves APP at Aβ amino acid 11 and a yet unknown enzyme cleaves at Aβ amino acid 5. βCTF can also be cleaved by α-secretase, resulting in a number of short Aβ peptides ranging from 14 to 16 amino acids in length, or by BACE2 (β2) at amino acids 20 or 34, adding to the number of different Aβ species found in CSF and cell culture media^6^.
