# Supplementary material for: Amyloid precursor protein expression and processing are differentially regulated during cortical neuron differentiation
Source: Sci Rep. 2016 Jul 7;6:29200. doi: 10.1038/srep29200 (PMC4935877; doi:10.1038/srep29200)
Supplement: Supplementary Information [file srep29200-s1.pdf]

Amyloid precursor protein expression and processing are differentially regulated during cortical neuron differentiation.

Petra Bergström[a,1], Lotta Agholme[a,1,*], Faisal Hayat Nazir[a], Tugce Munise Satir[a], Jamie Toombs[b], Henrietta Wellington[b], Joakim Strandberg[c], Thomas Olsson Bontell[c,d], Hlin Kvartsberg[e], Maria Holmström[a], Cecilia Boreström[f**], Stina Simonsson[f], Tilo Kunath[g], Anders Lindahl[f], Kaj Blennow[e], Eric Hanse[c], Erik Portelius[e], Selina Wray[b], Henrik Zetterberg[b,e]

Supplementary information

Supplementary Figure S1

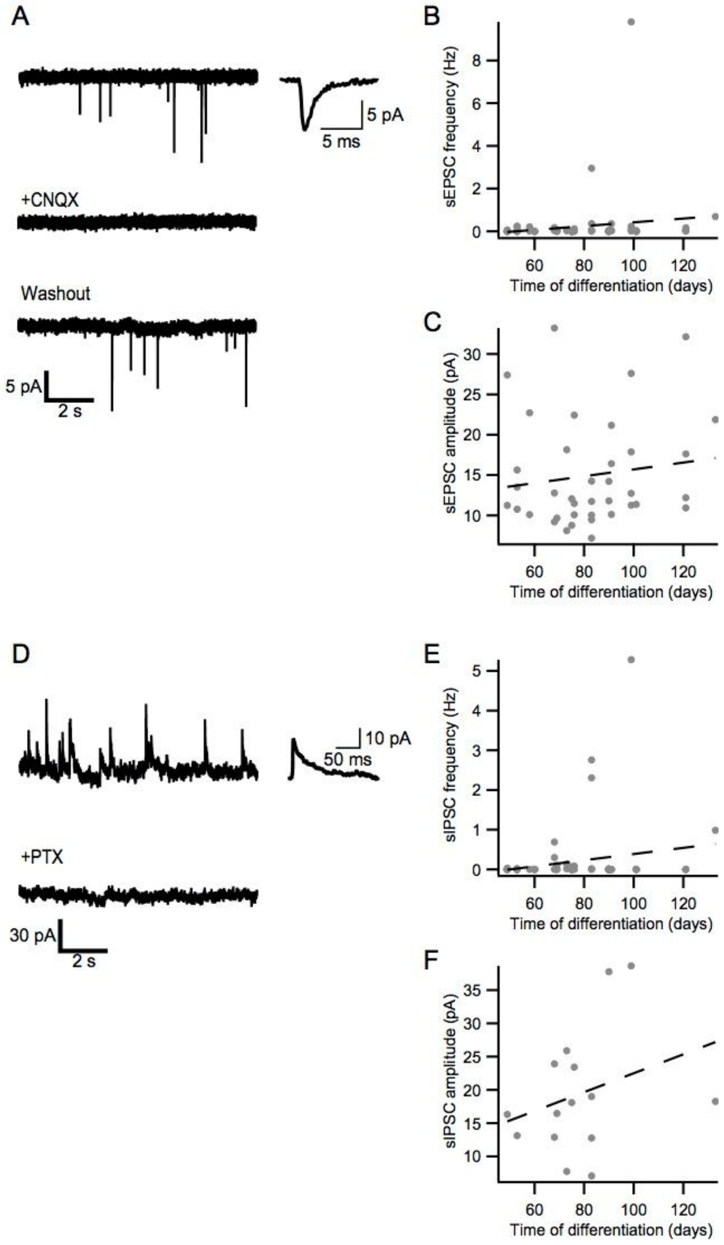

Supplementary Figure S1. Spontaneous synaptic activity onto iPSC-derived cortical neurons. (A) Example sweep of voltage-clamp recordings showing spontaneous AMPA evoked post-synaptic currents (EPSCs) recorded at -70 mV. These EPSCs were completely, and reversibly, blocked by the AMPA receptor antagonist CNQX. The average spontaneous AMPA EPSC is shown to the right. 38 out of 72 cells exhibited spontaneous AMPA EPSCs with average amplitudes ranging from 7.2 to 33 pA (n=38). (B) Graph summarizing spontaneous AMPA EPSC

frequency plotted versus time of differentiation (days), showing a significant positive relationship ($r=0.15$, $p<0.001$, $n=72$). (C) Graph summarizing average spontaneous AMPA EPSC amplitude plotted versus time of differentiation (days), showing a significant positive relationship ($r=0.14$, $p<0.001$, $n=38$). Cells not showing any spontaneous AMPA EPSC are not represented in this graph. (D) Example sweep of voltage-clamp recordings showing spontaneous GABA inhibitory post-synaptic currents (IPSCs) recorded at 0 mV. These IPSCs were completely blocked by the $GABA_A$ receptor channel open channel blocker picrotoxin (PTX). The average spontaneous GABA IPSC is shown to the right. 15 out of 61 cells exhibited spontaneous GABA IPSCs with average amplitudes ranging from 7 to 39 pA ($n=15$). (E) Graph summarizing spontaneous GABA IPSC frequency plotted versus time of differentiation (days), showing no significant relationship ($r=0.19$, $p>0.05$ $n=61$). (F) Graph summarizing average spontaneous GABA IPSC amplitude plotted versus time of differentiation (days), showing a significant positive relationship ($r=0.30$, $p<0.01$, $n=15$). Cells not showing any spontaneous GABA IPSC are not represented in this graph. The best fit to the data points in B, C, E and F was calculated using linear regression (dashed lines). The significance of the slope was determined using Student's t-test with n-2 degrees of freedom.

Supplementary Figure S2

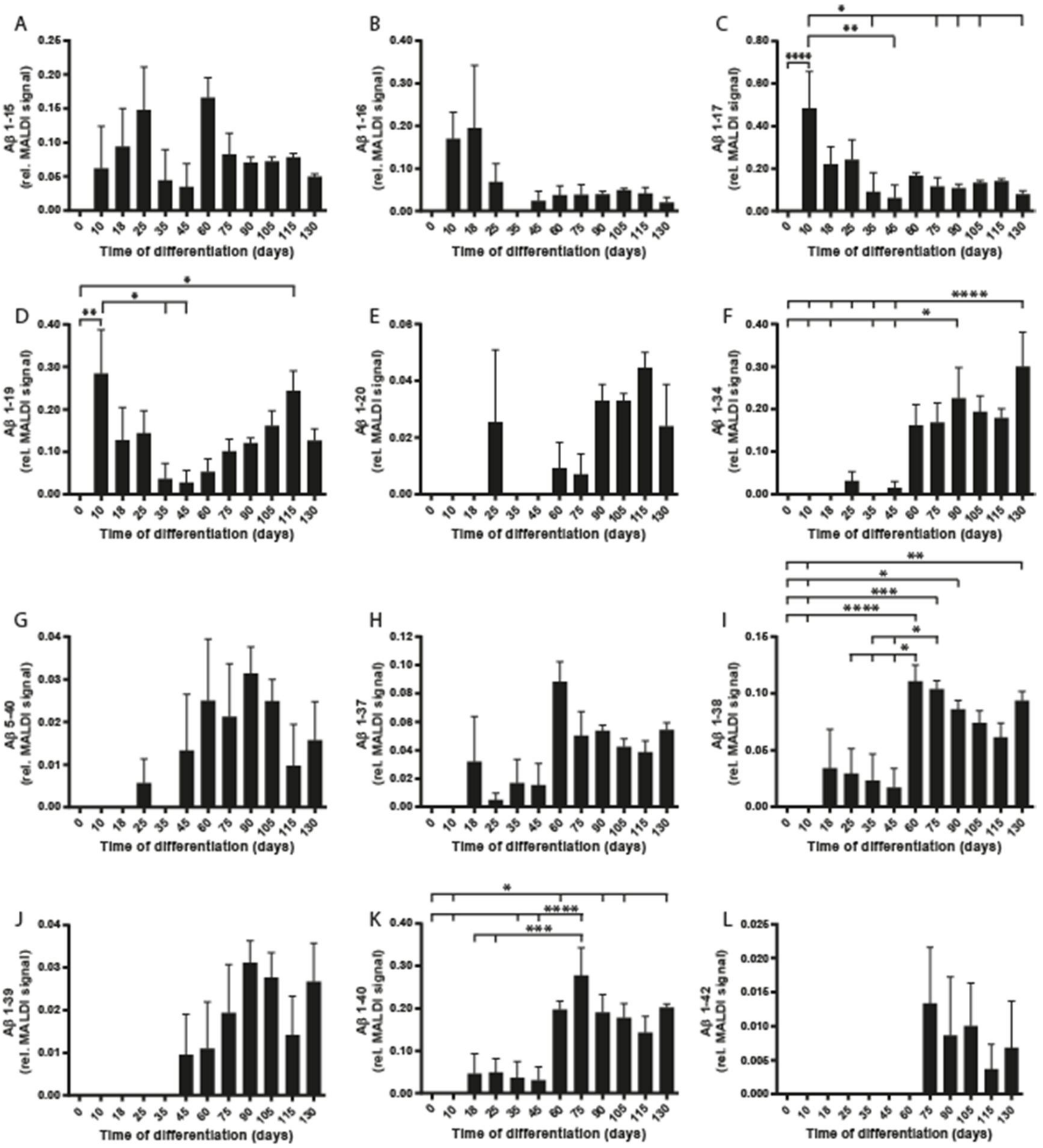

Supplementary Figure S2. Relative levels of all peptides detected in cell culture media at any stage during differentiation of iPSCs towards cortical neurons. Conditioned cell culture media was collected throughout differentiation of human iPSCs towards cortical neurons and secreted Aβ peptides were detected using IP-MS. In total, 12 Aβ peptides are detected in the cell media. No secretion of Aβ peptides is

detected from undifferentiated iPSCs (day 0). (A) Secretion of Aβ1-15 is detected from day 10, with a tendency to higher relative levels on days 25 and 60. (B) Maximal relative levels of Aβ1-16 are seen on days 10-18, to thereafter decrease. (C) A significant increase in Aβ1-17 is observed on day 10 compared with day 0, and a significant decrease on days 35-45/75-105/130 compared with day 10. (D) Aβ1-19 is significantly higher on days 10 and 155 compared with day 0 and significantly lower on days 35-45 compared with day 10. (E) Secretion of Aβ1-20 is detected on day 25, and thereafter on days 60-130. (F) High relative levels of Aβ1-34 are detected from day 60, reaching statistical difference on days 90 and 130 as compared to days 0-18/ 35-45 and days 0-45 respectively. (G) Secretion of Aβ5-40 is detected on day 25 and days 45-130, with a tendency to peak around day 90. (H) Secretion of Aβ1-37 is detected from day 18 of differentiation and peaks around day 60. (I) Secretion of Aβ1-38 is detected at low relative levels between days 18-45. Relative levels of Aβ1-38 were significantly higher on day 60 compared with days 0-10 and 25-45, on day 75 compared with days 0-10 and 35-45 and on days 90 and 130 compared with days 0-10 (J) Secretion of Aβ1-39 is first detected on day 45 and remains detectable until the end of differentiation. (K) Secretion of Aβ1-40 is first detected on day 18 and remains at low relative levels until day 45 of differentiation. Significantly higher relative levels are observed on day 60, 90, 105 and 130 compared with days 0-10. The highest relative levels are detected on day 75, when the levels differ statistically as compared to days 0-45. (L). Secretion of Aβ1-42 is first detected on day 75 and remains detectable throughout differentiation. Bars represent mean AUC for each respective Aβ peptide related to the total AUC of all 12 identified Aβ peptides in the same sample +/- SEM, n=4. $*=p\leq0.05$, $**=p\leq0.01$, $***=p\leq0.005$, $****=p\leq0.001$.
